# Impaired B cells survival upon production of inflammatory cytokines by HIV-1 exposed follicular dendritic cells

**DOI:** 10.1186/s12977-016-0295-4

**Published:** 2016-09-05

**Authors:** Farideh Sabri, Alejandro Prados, Raquel Muñoz-Fernández, Rebecka Lantto, Pablo Fernandez-Rubio, Aikaterini Nasi, Sylvie Amu, Jan Albert, Enrique Garcia Olivares, Francesca Chiodi

**Affiliations:** 1 Department of Microbiology, Tumor and Cell Biology, Karolinska Institutet, Nobels väg 16, 17177 Stockholm, Sweden; 2 Unidad de Immunologia, Instituto de Biopatologia y Medicina Regenerativa, Complejo Hospitalario Universitario de Granada, Granada, Spain; 3 Department of Clinical Microbiology, Karolinska University Hospital, Stockholm, Sweden

**Keywords:** Follicular dendritic cells, HIV-1, Chemokines, Inflammation, B cells

## Abstract

**Background:**

Follicular dendritic cells (FDCs) are important components in the organization of germinal centers in lymphoid tissue where, following antigen presentation, B cells differentiate into memory B cells. The possibility of establishing primary cell lines from FDCs isolated from lymphoid tissue paved the way for characterization of FDC biological properties. We exposed primary FDC cell lines to HIV-1 strains in vitro and studied changes in the chemo-attractive properties of FDCs and release of inflammatory cytokines.

**Results:**

FDC lines expressed several known and putative HIV-1 receptors; viral genome was amplified in HIV-1 exposed FDCs which released low levels of p24 HIV-1 protein in culture supernatants, but were not definitely proven to be productively infected. Exposure of FDCs to HIV-1 strains did not change the expression of markers used to characterize these cells. HIV-1 exposed FDCs, however, changed the expression of chemo-attractants involved in cell recruitment at inflammatory sites and increased the production of several inflammatory cytokines. The inflammatory milieu created upon HIV-1 exposure of FDCs led to impaired B cell survival in vitro and reduced Ig production.

**Conclusions:**

FDC lines exposed to different HIV-1 strains, although not able to support productive HIV-1 replication, show an increased production of inflammatory cytokines. Our in vitro model of interactions between HIV-1 exposed FDC lines and B cells suggest that exposure of FDCs to HIV-1 in vivo can contribute to inflammation within germinal centers and that this pathological event may impair B cell survival and contribute to impaired B cell responses during HIV-1 infection.

**Electronic supplementary material:**

The online version of this article (doi:10.1186/s12977-016-0295-4) contains supplementary material, which is available to authorized users.

## Background

Follicular dendritic cells (FDCs) are unique to primary and secondary lymphoid follicles and comprise the best defined stromal cell subset [1]. The origin of FDCs is uncertain; some studies suggest that these cells have stromal and mesenchymal origin and most likely emerge from ubiquitous perivascular precursors [2, 3] while another report, basing on the antigenic phenotype of FDCs, related their origin to bone marrow stromal cell progenitors and myofibroblasts [4].

FDCs are important components in the organization of the germinal centre (GC) microarchitecture through an elaborated physical interplay with B cells. The major function of FDCs within the GC microenvironment is to present unprocessed antigen in the form of immune complexes (ICs) to B cells and to provide chemokines, adhesion molecules and trophic factors to shape adaptive B cell responses. Stationary FDCs attract motile CXCR5^+^ B cells into the secondary lymphoid organs by providing them with B cell chemo-attractants including CXCL13 [2]. B cells, in turn, are essential for FDC development and maintenance through the production of lymphotoxins (LFs) and, to a lesser extent, tumor necrosis factor (TNF). The absence of B cells, or of either LTs or TNF in lymphoid organs results in lack of FDC formation, as demonstrated in murine models [5–7].

FDCs have a unique ability to trap ICs in B cell follicles over a long period of time [7, 8]. Presentation of antigen at the surface of FDCs is critical for clonal selection and affinity maturation of activated B cells. The FDC-IC complex stimulates cognate follicular B cells via the B cell receptor, and provides additional signals through the complement and FC receptors. The role that FDCs have in the generation of memory B cells has progressively become evident.

FDCs are major reservoirs for HIV-1 within the lymph nodes of infected patients [9, 10]. Highly infectious HIV-1 virions coated with HIV-1 antibodies are trapped on the processes of FDCs [11]. In addition to their role as important reservoirs of infectious virus, FDCs can contribute to increased HIV-1 production in the lymphoid tissue microenvironment through a soluble TNF-α mediated mechanism [12].

The expression of potential HIV-1 receptors on FDCs has been poorly characterized and the possibility that these cells may become the target of productive HIV-1 infection has not been conclusively ruled out. To achieve attachment to CD4+ T cells, HIV-1 binds primarily to its main receptor, the CD4 molecule, and to at least one of its co-receptors, the CXCR4 or CCR5 chemokine receptors. Several other molecules have, however, been shown to act as potential HIV-1 receptors in the context of HIV-1 infection of several cell types in vitro but their significance in vivo is uncertain. The HIV-1 envelope protein gp120 can bind to the activated form of the integrin α_4_β_7_ on CD4+ T lymphocytes [13]. Moreover HIV-1 has the ability to attach to the Sialic acid-binding Ig-like lectin 1 (Siglec-1), the TAM receptors on dendritic cells (DCs) and the DC specific ICAM-3 grabbing non integrin (DC-SIGN) on DCs and B-cells [14–16]. The critical role of CD21 in HIV-1 trapping by cells in the lymph nodes was shown in a murine study [17].

Several phenotypic and functional abnormalities have been described to affect B cells during HIV-1 infection. The frequency of resting memory B cells is reduced in the peripheral blood of HIV-1 infected adults and children [18–20] and antiretroviral therapy initiated in patients with chronic HIV-1 infection does not restore the physiological levels of memory B cells [21]. The reduced number of resting memory B cells may be one of the underlying causes for the poor response to vaccines and declined serological memory in HIV-1 infected individuals [22].

FDCs play an important role for instructing the molecular contact between T and B cells within the GCs and it is therefore reasonable to ask whether B cell dysfunctions during HIV-1 infection may be initiated in the context of GC formation. In monkeys infected with simian immunodeficiency virus (SIV) and rapidly progressing to disease, disruption of the FDC network in the GCs could be monitored early following infection, together with progressive depletion of proliferating B cells [23]. Interestingly, no obvious depletion of peripheral CD4+ T cells was observed in these monkeys while a high frequency of productively SIV infected cells and large amounts of accumulated viral RNA were evident in the GCs [23].

For many years it has been difficult to conduct in vitro studies with FDCs, partly because of the difficulty in obtaining a large number of these cells which exists at a low proportion in lymphoid tissues, representing only 1 % of cells in this compartment. Another limiting factor in establishing FDC cultures from lymphoid tissue is the presence of B cells tightly entrapped in FDC dendritic processes. The definition of immune alterations which may take place in the FDCs as result of HIV-1 infection require additional studies. In the present work we exposed primary FDC lines [4] to HIV-1 in vitro and studied whether, upon this condition, changes in the biology of FDCs took place which could affect migration and survival of B cells, mimicking events which commonly take place in the GC between FDCs and B cells. The results show that FDC lines are unlikely to be directly infected and to support HIV-1 replication. HIV-1 exposed FDCs, however, changed the expression of chemo-attractants and increased the production of several inflammatory cytokines, conditions which caused impaired B cell survival and declined Ig production.

## Results

### The phenotype of HIV-1 exposed FDCs is comparable to non-exposed cells

We aimed at studying whether exposure of FDCs to HIV-1 could modify the antigenic profile of FDCs. For the purpose, four FDC lines (1401, 1402, 1403, 8–13) were characterized for phenotypical markers previously associated with FDCs [4]: CD10, CD14, CD31, CD35, CD45, CD54 or ICAM-1, CD90, CD106 (or VCAM-1), CD140B, CD146, ASMA, HLA-DR, MFGE8 and Podoplanin. In addition, the expression of molecules involved in the interaction of FDCs with B and T-cells, including CD44 [24], CD105 [25], CXCL12 [26] and CXCL13 [27] were also assessed on FDCs. The gating strategy for selection of CXCL12 and MFGE8 positive cells in the FDC line 1403, exposed or not to HIV-1 strains IIIB and SF162, is shown in Additional file 1: Figure S1.

**Fig. 1:**
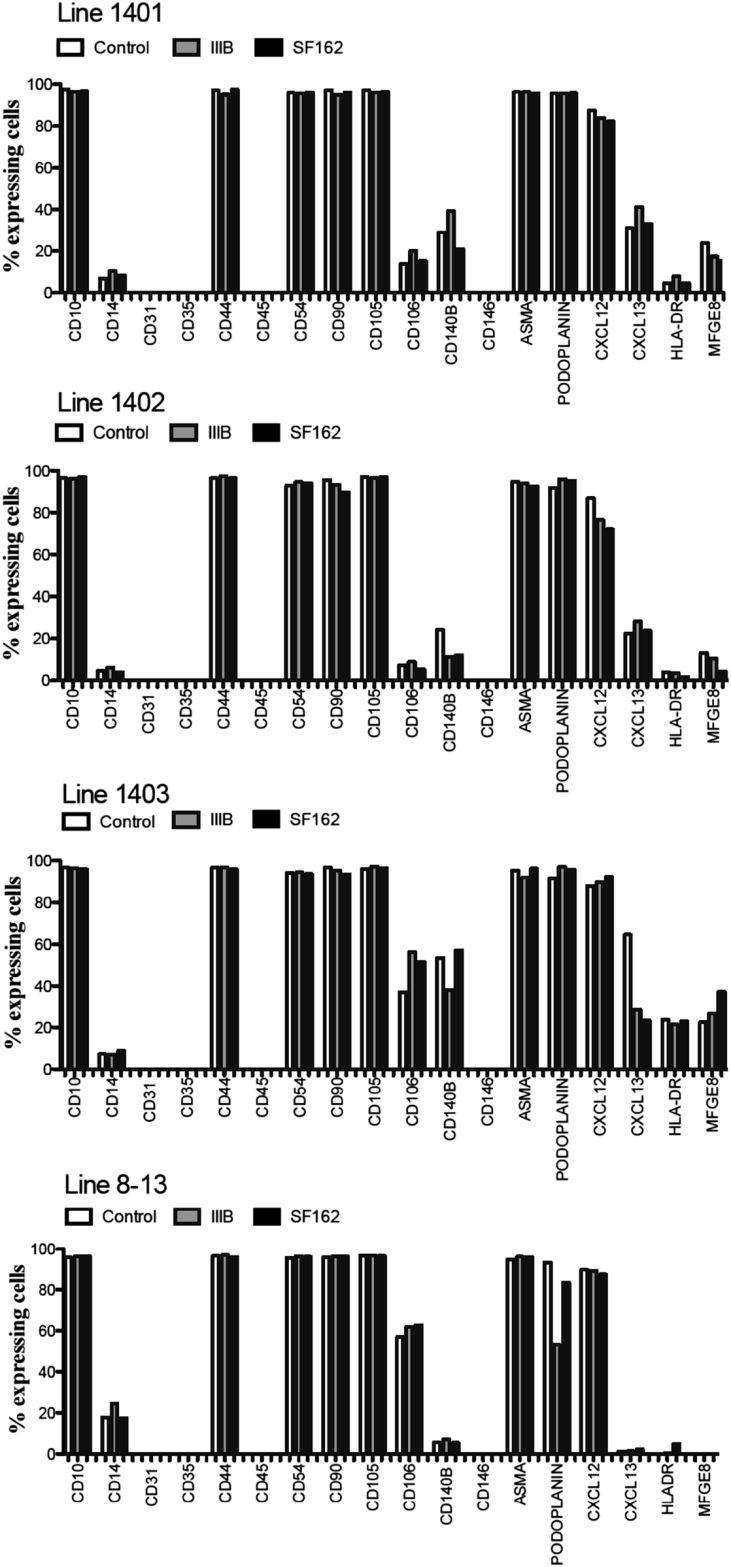
Expression of surface markers on HIV-1 exposed FDCs. The expression of characteristic FDC markers was measured by flow cytometry in FDC lines 1401, 1402, 1403 and 8–13 at 3 days post exposure to either IIIB or SF162 HIV-1 strains, or control medium. *Bars* represent the percentage of positive cells in FDC lines. Exposure to HIV-1 did not change significantly the phenotypic characteristics of FDCs

FDC cultures exposed for 3 days to the HIV-1 strains IIIB and SF162, or to medium, showed comparable expression of CD10, CD14, CD44, CD54, CD90, CD105, CD106, CD140B, CXCL12, ASMA, HLA-DR and MFGE8 (Fig. 1). The expression of HLA-DR and MFGE8 remained stable after HIV-1 exposure but differed between the individual FDC lines (Fig. 1). The expression of podoplanin and CXCL13 remained stable in 3 of the 4 lines analyzed following exposure to HIV-1 strains (lines 1401, 1402, and 1403 for podoplanin and lines 1401, 1402 and 8–13 for CXCL13). However in the FDC line 1403, CXCL13 expression was reduced following exposure to HIV-1 strains IIIB and SF162 (from 65 to 29 and 23 %); in addition the expression of podoplanin was reduced once the cells were exposed to IIIB strain, but not when the cells where exposed to SF162 (from 93 to 53 and 84 %). The expression of CD31, CD35, CD45 and CD146 was maintained stably negative following exposure to HIV-1 in all four lines as shown in Fig. 1. Taken together, these results show that exposure to HIV-1 does not alter significantly the phenotypical characteristic of FDCs in vitro.

### Exposure of FDC lines to HIV-1 strains

**Fig. 2:**
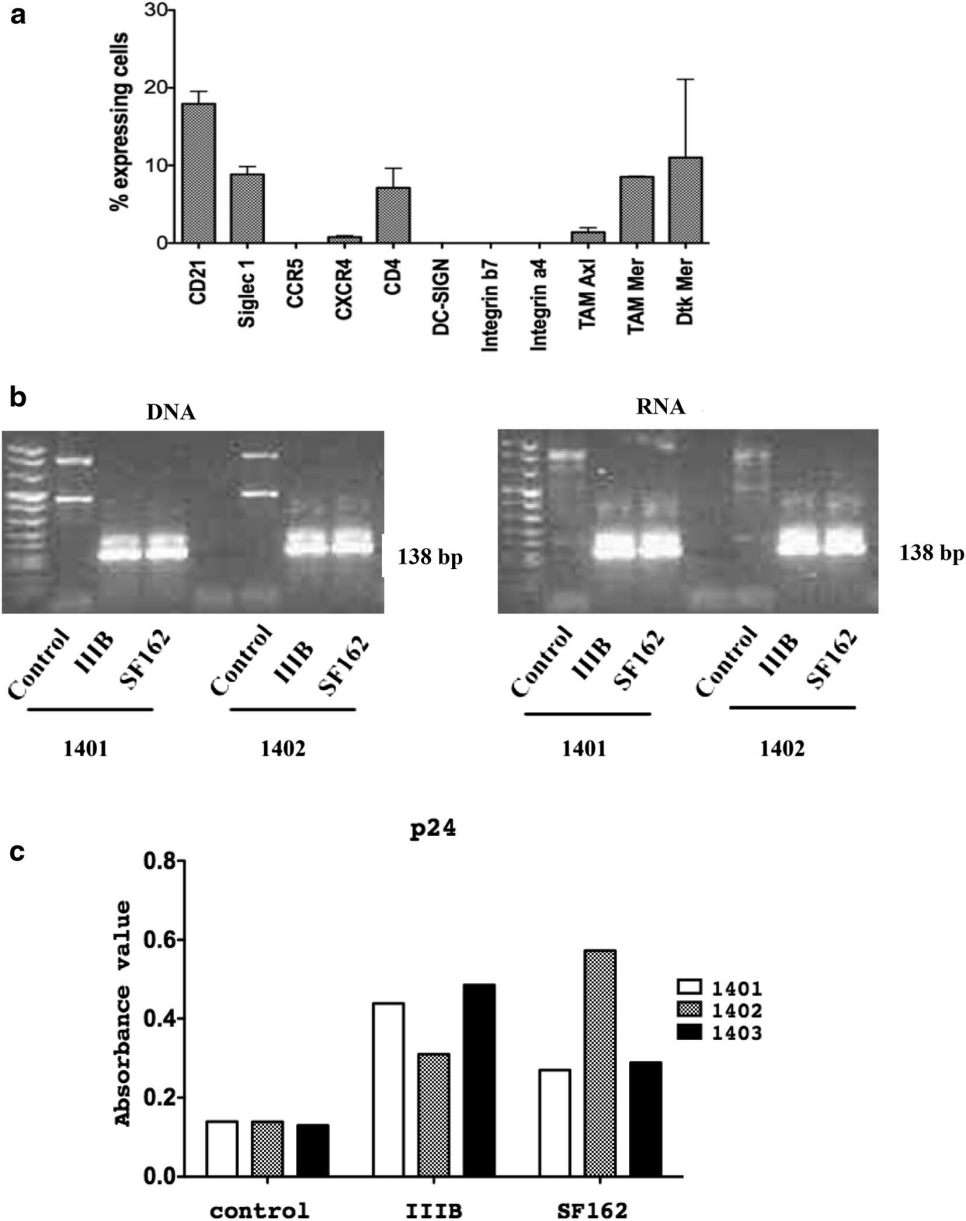
Exposure of FDC lines to HIV-1 strains. Expression of potential HIV-1 receptors on FDC lines (**a**). The *bars* represent the mean expression value and standard deviation for CD21, Siglec 1, CCR5, CXCR4, CD4, DC-SIGN, β7 and α4 integrins, TAM Axl, TAM Mer and Dtk Mer in 3 FDC lines. Data was normalized to the percentage of positive cells detected with the isotype control antibodies. Nested PCR for detection of HIV-1 RNA and proviral DNA (**b**). The expected PCR product size of 138 bp detected through pol primers JA79-JA82 and JA80-JA81 confirmed the infection of FDC 1401 and 1402 cells with the HIV-1 strains IIIB and SF162. The upper band visible in the picture represents the amplicon for the outer primers. RNA and DNA were prepared from FDCs cells at day 7 post-exposure. HIV-1 p24 antigen in culture supernatants from FDC lines 1401, 1402 and 1403 at 10 days post-exposure with IIIB and SF 162, as measured by ELISA (**c**). The cut off OD value is 0.28 and results above this limit where considered positive

Three primary FDC lines (8–13, 9–13 and 10–13) were characterized for the expression of potential HIV-1 receptors and co-receptors. Flow cytometry analysis demonstrated the consistent expression of several potential HIV-1 receptors on FDCs cells: CD4 (expressed on 7.1 % ± 5 of FDCs), CD21 (17.9 ± 3.2), Siglec 1 (8.8 % ± 2), TAM Axl (1.4 % ± 0.8), TAM Mer (8.5 % ± 0.09), Dtk Mer (11 % ± 14.2), low expression of CXCR4 (0.78 % ± 0.35) and no expression of the two components of the α4β7 Integrin, DC-SIGN and CCR5 (Fig. 2a). The gating strategy for detection of CD4 and CCR5 molecules on the 9–13 line is shown in Additional file 2: Figure S2.

The interaction of HIV-1 with FDCs has been described to be limited to capture of the virus by FDCs through immune complexes; whether HIV-1 can directly infect and replicate in FDCs has been poorly studied. HIV-1 pol sequences were detected in DNA and RNA extracted from FDCs exposed for 7 days to IIIB or SF162 HIV-1 strains, but not in cells cultured in medium (Fig. 2b).

Low levels of HIV-1 p24 were detectable in the supernatant of all three FDCs lines exposed to HIV-1 for 10 days as compared to the non-exposed lines. The p24 absorbance values detected by ELISA were low but above the cut-off absorbance value of 0.28 (Fig. 2c). Virus was detected in the supernatants of IIIB exposed FDCs 1401 and 1403 (absorbance 0.44 and 0.48) and in the SF162 exposed FDC line 1402 (absorbance 0.57).These observations suggest that a low productive HIV-1 infection may take place in FDCs in vitro.

**Fig. 3:**
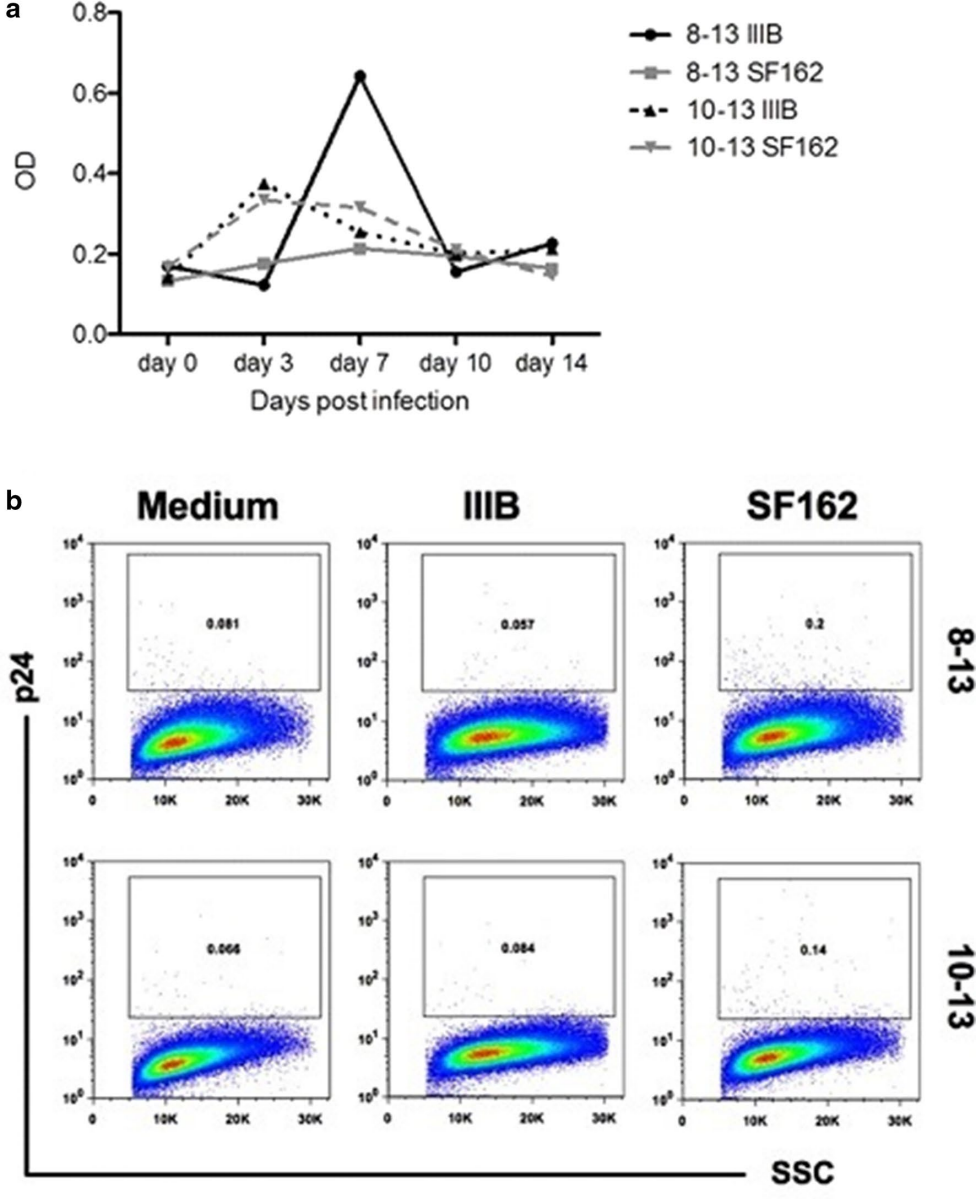
Kinetics of p24 production in FDC lines exposed to HIV-1 and intracellular p24 staining. The FDC lines 8–13 and 10–13 were exposed to the HIV-1 strains IIIB and SF162 over-night. Thereafter the virus was removed from the cultures which were washed extensively with tissue culture medium; 6 h following the last washing, the time point 0 was collected. Supernatants were also collected at days 3, 7, 10 and 14. **a** Detection of HIV-1 p24 protein in culture supernatants at different days of culture. In **b** detection of p24 intracellular antigen in cell lines 8–13 and 10–13 at 1 week after exposure to IIIB and SF162 HIV-1 strains is shown. The p24 positive cells are shown inside the gate on the *dot-plots*

In order to further study if FDC cell lines were productively infected we performed kinetics experiments of p24 release into culture supernatants (Fig. 3a) and HIV-1 RNA (not shown) and also stained FDC cells for intracellular p24 production (Fig. 3b). Production of p24 in culture supernatant of FDC lines 8–13 and 10–13 exposed to IIIB and SF162 isolates was followed for 2 weeks; the results of this experiment showed that a minimal level of p24 production could be detected in cultures exposed to the two HIV-1 strains between 3 and 7 days post-infection (Fig. 3a). Intracellular p24 detection at 7 days post-infection showed a similar low number of p24 positive cells in 8–13 and 10–13 FDC cultures exposed to strains IIIB and SF162 as compared to cultures grown in medium (Fig. 3b).

HIV-1 RNA was quantified in supernatants of FDC lines 8–13 and 10–13 exposed to IIIB and SF162 at day 0 of infection and at 7 days post-infection. The specimens at day 0 were collected after over-night incubation of the virus with FDC cultures, following extensive washings. A semi-quantitative PCR method revealed that HIV-1 RNA was detectable in culture supernatants at dilutions between 1:10 and 1:100 (results not shown) and that the RNA levels did not significantly increase between days 0 and 7 from infection. These experiments support the scenario that only few HIV-1 infected cells are present in FDC cell cultures exposed to HIV-1 strains and that the frequency of infected cells does not increase over-time.

Altogether the experiments indicate that HIV-1 strains do not establish a clearly detectable productive infection in FDC lines. For this reason, we will use the terminology “exposed” when referring to FDC lines incubated with virus.

### mRNA expression of chemokines and chemokine receptors in HIV-1 exposed FDCs

**Fig. 4:**
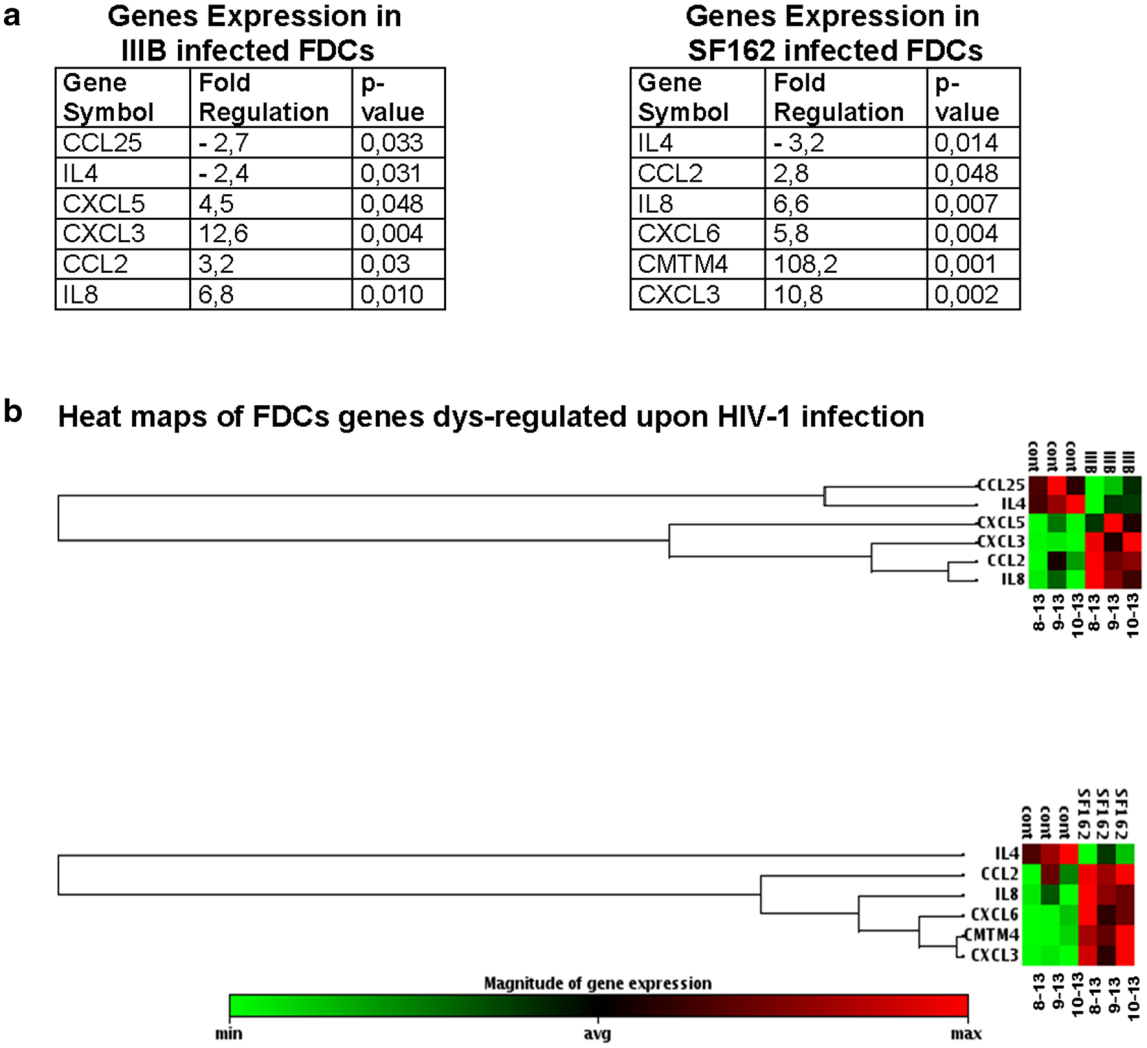
Dysregulated expression of chemokine genes in HIV-1 exposed FDCs. A summary of the genes whose expression was significantly altered in FDCs exposed to either IIIB or SF162 is presented in **a**. Genes whose expression was significantly (p < 0.05) up- or down-regulated more than twofolds are shown in the table. The results are also presented as heat maps (**b**)

To study whether HIV-1 infection impacts on the chemotactic and migratory capacity of FDCs cells, we analyzed mRNA expression of chemokines and chemokine receptors in IIIB and SF162 exposed and control FDCs. We analyzed 84 genes that encode for human chemokines and chemokine receptors in FDC lines 8–13, 9–13 and 10–13 at day 2 post-virus exposure. Figure 4 illustrates, both in fold changes and as heat maps, the genes which expression was dysregulated upon HIV-1 infection. A significant up-regulation (>2 fold changes, p < 0.05) of genes encoding for CCL2, CXCL3 and IL-8 was observed in the FDC lines exposed to both IIIB and SF162 HIV-1 strains (Fig. 4a, b), as compared to FDC lines treated with culture medium. The gene expression of the chemokine ligand CXCL5 was up-regulated only in IIIB exposed FDCs, while an increased expression of CMTM4 and CXCL6 genes was revealed in SF162 exposed cells (Fig. 4). Among the down-regulated genes, the expression level of CCL25 was decreased more than twofold (p < 0.05) in SF162 exposed cells while a down-regulation of IL-4 gene expression was detected in both IIIB and SF162 exposed FDCs.

**Fig. 5:**
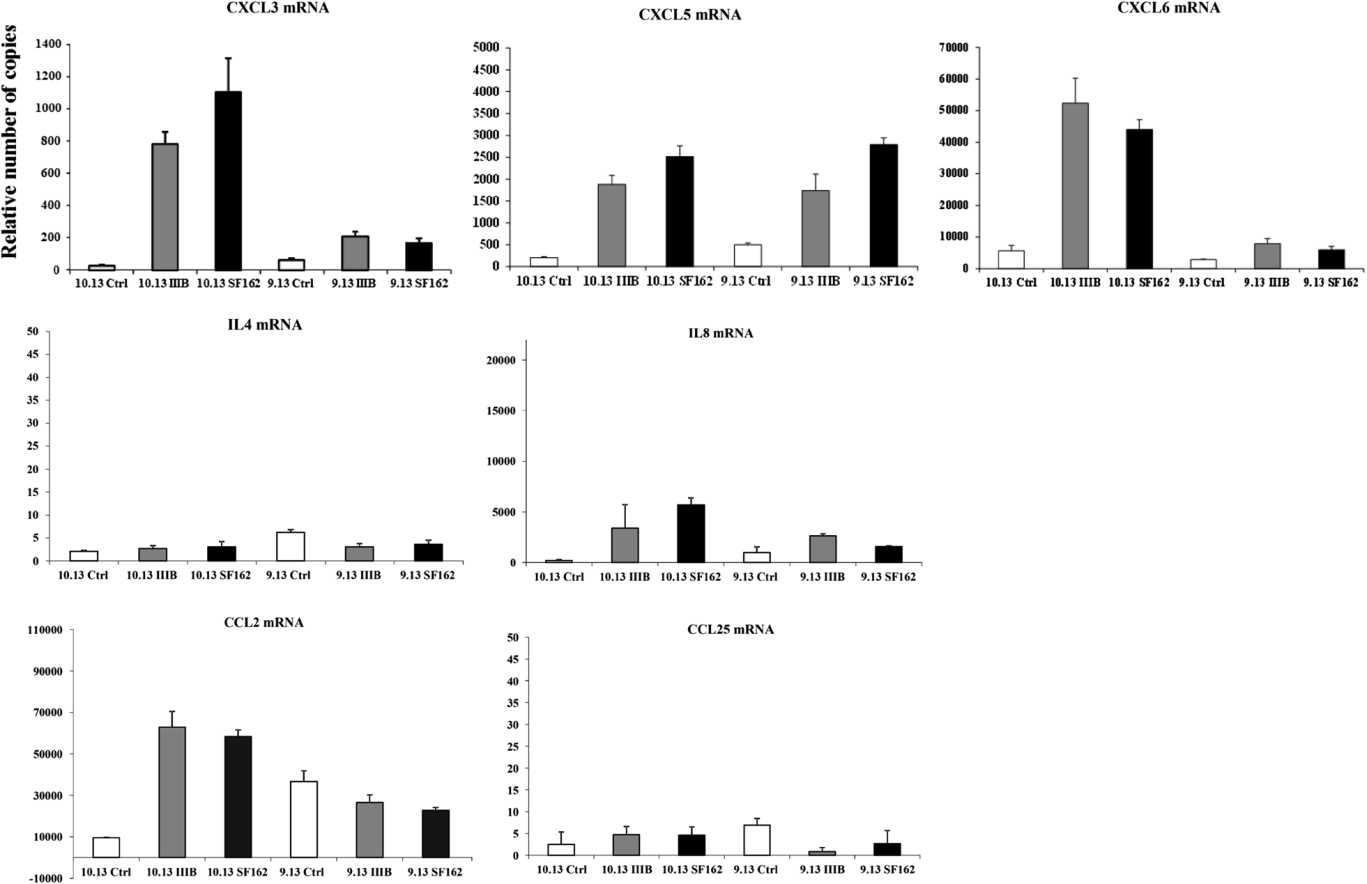
Real-time PCR of control and HIV-1 exposed FDC lines. Real-time PCR was performed to verify the dys-regulated expression of genes revealed by gene array. The genes analysed by RT-PCR comprise CXCL3, CXCL5, CXCL6, IL-4, IL-8, CCL2 and CCL25. The numbers on the *y axis* show the relative number of copies for the chemokine gene. The samples were normalized by quantitation-relative curve with human beta-actin

In order to further verify the results obtained by microarray we used real-time PCR. The results from real-time PCR could, to a large extent, verify the microarray results. Control and HIV-1 exposed FDCs were studied for the expression of pro-inflammatory chemokines including CXCL3, CXCL5 and CXCL6 (Fig. 5). These chemokines were up-regulated in the HIV-1 exposed FDCs; CXCL5 had a similar expression pattern in both 9.13 and 10.13 lines while the expression of CXCL3 and CXCL6 in 10.13 FDCs was higher than in the 9.13 FDC line.

IL-8 expression was up-regulated in HIV-1 exposed FDCs with high positive modulation in SF162. In relation to IL-4, there was no expression and modulation in any line (control and HIV-1 exposed FDCs).

The expression of the chemokines CCL2 and CCL25 was different in the different HIV-1 exposed lines: the 10.13 FDC line showed an increased expression for both chemokines whereas the opposite pattern was shown for the 9.13 FDC line.

### Chemo-attractive activity of FDCs and antigen presenting capacity following exposure to HIV-1 strains

To evaluate the impact that HIV-1 may have on the properties of FDCs to release chemo-attractants for B and T-cells, migration of T and B cells towards cell culture supernatant from HIV-1 and medium exposed FDCs was evaluated by trans-well migration assay.

PBMCs from three healthy donors were activated for 2 days with CpG, PHA or the combination of IgM and anti-CD40; the activation of B and T cells was monitored through the expression of CD69 activation marker (Additional file 3: Figure S3). Thereafter, the PBMCs were cultured for 4 h in trans-well membrane plates in presence of supernatant obtained from FDC cultures 7 days post-HIV-1 exposure. Migration of T and B cells was measured by counting the migrated cells and subsequent calculation (by flow cytometry) of the frequency of B and T cells within the migrated cells.

The activation experiment revealed a varying degree of CD69 expression (Additional file 3: Figure S3A–C) on PBMCs, B and T cells. CPG, PHA and the combination of IgM and anti-CD40 were all effective, although to a different degree, in upregulating the expression of CD69 on B cells from three donors (Additional file 3: Figure S3B) as the expression of CD69 ranged between 15 and 82 % for the different activation signals. In T cells, upregulation of CD69 occurred mostly with PHA (Additional file 3: Figure S3C).

**Fig. 6:**
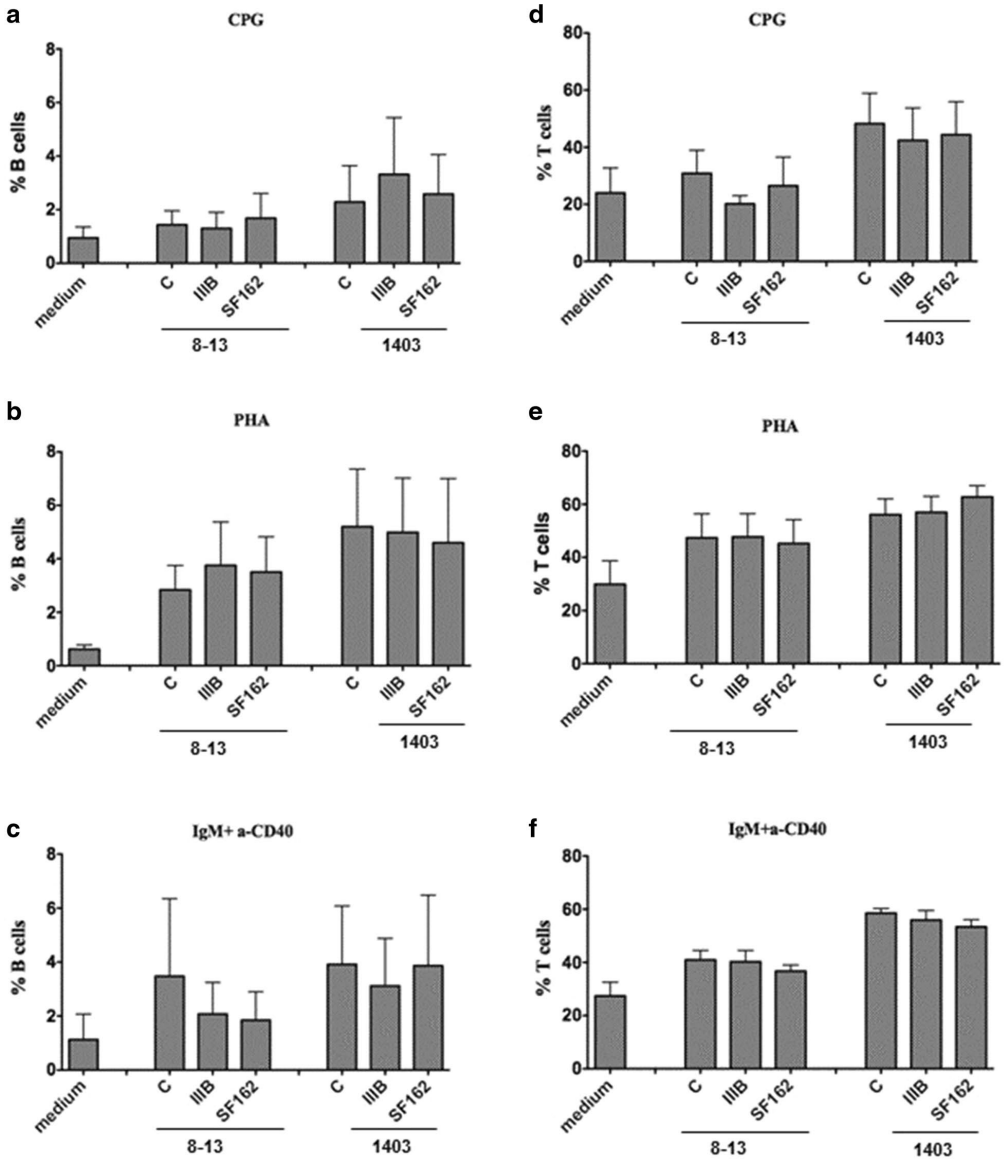
Migration of B and T cells towards HIV-1 exposed FDCs. PBMCs from 3 donors were activated with CpG, PHA or the combination of anti IgM and anti CD40 and placed for 4 h on trans-well chambers containing the supernatant from FDCs (8–13 and 1403) exposed to HIV-1 (IIIB or SF162 strain) for 7 days. Cells were also migrated towards medium. The frequency of migrated CD19+ B (**a**–**c**) and CD3+ T-cells (**d**–**f**) in total migrated cells were measured by Flow cytometry. The data represent the cumulative mean and standard deviation for 3 activation conditions in cells from 3 donors

The frequency of B and T cells among PBMCs migrating towards the supernatants from HIV-1 exposed (IIIB and SF162) FDC lines is shown in Fig. 6a–f. The frequencies of migrated cells reflected the composition of PBMCs; no significant differences in the frequency of migrated B and T-cells towards the culture medium from HIV-1 exposed FDCs and control supernatant were observed (Fig. 6a–f).

The antigen capturing capacity of FDCs is an important functional property of these cells; this feature was evaluated in FDCs exposed to HIV-1 for 1 day by culturing cells for 4 h with Alexa Fluor 488 conjugated ovalbumin. The relative MFI of ovalbumin capturing in cells cultured with medium was 0.85 ± 0.07, for IIIB exposed cells 0.78 ± 0.15 and for SF162 exposed cells 0.83 ± 0.12. These results demonstrated that FDCs exposed to HIV-1 for 24 h maintained an intact antigen capturing capacity (Additional file 4: Figure S4).

### Production of inflammatory cytokine by HIV-1 exposed FDCs

The production and secretion of cytokines including IL-1α, IL-1β, IL-2, IL-4, IL-6, IL-8, IL-10, IL-12, IL-17A, IFN-γ, TNF-α and GM-CSF was evaluated by ELISA-array in the culture supernatant of two HIV-1 exposed FDC lines (1401 and 1402) and non-exposed cultures.

**Fig. 7:**
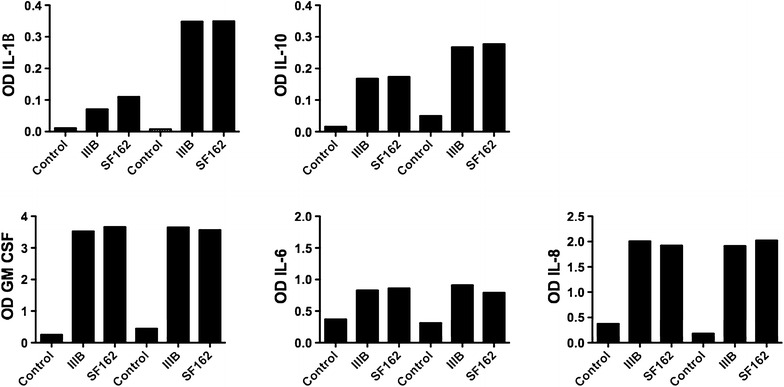
Cytokine production from FDCs upon HIV-1 infection. The FDC lines 1401 and 1402 were exposed to HIV-1 strains IIIB and SF162 overnight, washed and cultured for 10 days. Cytokines release in supernatant were measured by ELISA array. The data represent the absorbance value of samples after subtraction of the value for the negative control

IL-1β, IL-2, IL-4, IL-12, IL-17A, IFN-γ and TNF-α were not detectable in the supernatants of either HIV-1 or medium exposed FDCs (results not shown). Interestingly, the production of the inflammatory cytokines IL-6, IL-8, IL-1β and GM-CSF increased in HIV-1 exposed cultures (Fig. 7) with GM-CSF and IL-8 increasing mostly. These observations indicate that HIV-1 exposure of FDCs can induce the production of cytokines involved in inflammation and DC maturation. Interestingly, also the levels of production of IL-10, an anti-inflammatory cytokine, were elevated in FDC cultures exposed to HIV-1 strains.

**Fig. 8:**
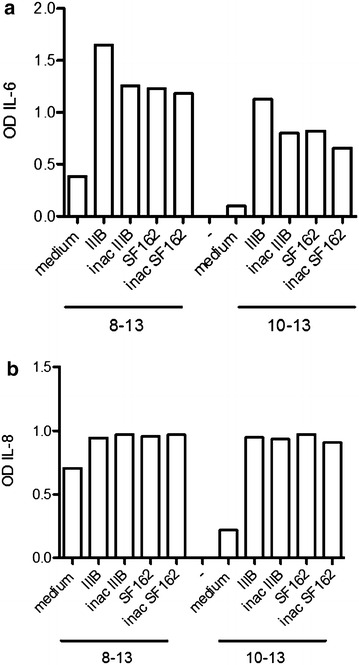
Cytokine production from FDC lines exposed to inactivated virus. The FDC lines 8–13 and 10–13 were exposed to virus strains IIIB and SF162 in their native state and following inactivation at 56 °C for 60 min. At 10 days from virus exposure, supernatants from FDC cultures were collected and analyzed for IL-6 (**a**) and IL-8 (**b**) production

In order to study if HIV-1 replication in exposed cultures was essential for production of inflammatory cytokines IL-6 and IL-8, we heat-inactivated (56 °C for 60 min) the virus strains IIIB and SF162; FDC lines 8–13 and 10–13 were thereafter exposed to heat-inactivated viruses in parallel with the native virus strains. Supernatants from FDCs cultures exposed to native and heat-inactivated viruses were collected at 10 days post-infection and the levels of IL-6 and IL-8 measured. As shown in Fig. 8a, b the levels of IL-6 and IL-8 are comparable in supernatants from cultures exposed to native and heat-inactivated viruses. Although it cannot be excluded that replication competent viruses may remain in the heat-inactivated virus preparations, the experiment strongly suggests that virus components may have a role in inducing production of IL-6 and IL-8 through a mechanism which is not yet characterized.

### B cells die when co-cultured with HIV-1 exposed FDCs

In order to understand the impact that the inflammatory environment created by the production of inflammatory cytokines from HIV-1 exposed FDCs could have on T and B cells we cultured HIV-1 exposed FDCs with PBMCs obtained from healthy donors. The FDC lines 1401 and 1402 were exposed to HIV-1, washed extensively and cultured for 2 days before addition of PBMCs from non-infected donors; these mixed culture of FDCs and PBMCs were prolonged for additional 2 and 7 days. Following these days of co-culture, the PBMCs were analyzed by flow cytometry for viable lymphocytes and frequency of B and T-cells, while the cell free culture supernatants were used for IgG and IgM detection by ELISA.

**Fig. 9:**
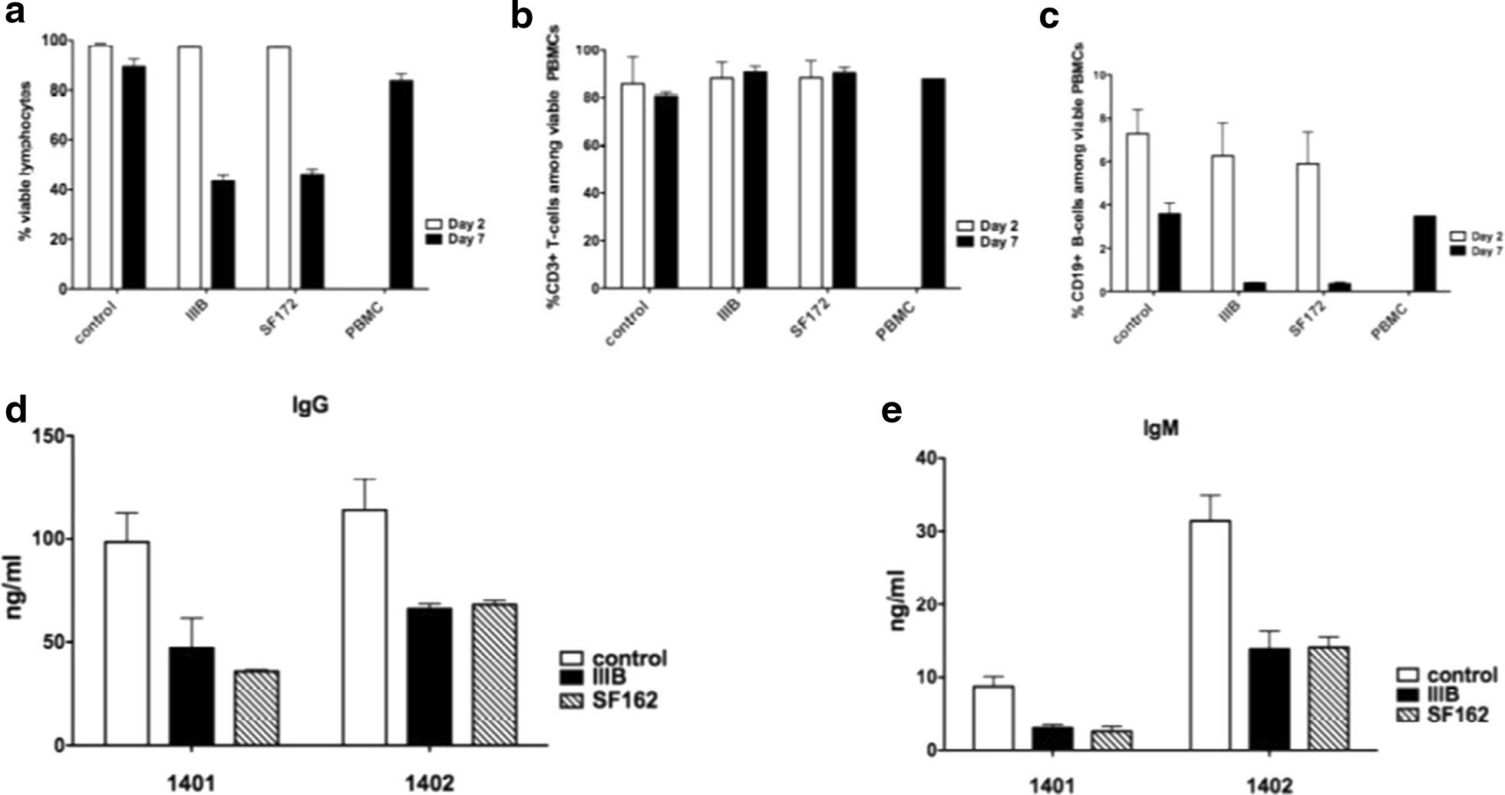
Viability and cell death in lymphocytes co-cultured with HIV-1 exposed FDCs. The FDC lines 1401 and 1402 were exposed to HIV-1 IIIB or SF162 o.n., washed and cultured for 2 days before PBMCs from healthy blood donors were added. The viability of lymphocytes was evaluated at 2 and 7 days of co-cultivation by Flow cytometry. The data represent the cumulative mean of vivid (**a**) negative lymphocytes in PBMCs from three donors co-cultured for either 2 or 7 days with FDCs. The viability of T (**b**) and B (**c**) cells is presented as cumulative mean and SD of vivid negative CD3+ and CD19+ positive cells in PBMCs from three donors co-cultured for either 2 or 7 days with FDCs. The levels of IgG (**d**) and IgM (**e**) in supernatants from PBMCs co-cultured with HIV-1 exposed and control FDCs for 10 days and measured by ELISA

The viability of lymphocytes co-cultured with HIV-1 exposed FDCs was not affected at 2 days of co-culture whereas was markedly reduced at 7 days declining from 83 % in non-exposed cultures to 40 % in IIIB exposed and 41 % in SF162 exposed FDCs (Fig. 9a).

The frequency of viable CD3+ T cells was not changed at either day 2 or 7 when comparing PBMCs cultured with control or HIV-1 exposed FDCs (Fig. 9b). The frequency of CD19+ cells at day 2 of co-culture was comparable among PBMCs cultured with either HIV-1 exposed or medium FDCs. On the other hand, the frequency of CD19+ B cells was reduced from 3.6 % in the presence of control FDC lines to 0.39 % in IIIB and 0.36 % in SF162 exposed FDCs 7 days after co-cultivation (Fig. 9c). The frequency of CD19+ cells among PBMCs cultured for 7 days in absence of FDCs was similar to the frequency of B cells among PBMCs cultured in presence of control FDCs (3.5 %). These results indicate that HIV-1 exposed FDCs may exert a pathological specific impact on B cells, by inducing cell death in co-cultures of FDC lines and PBMCs.

In order to further dissect the biological relevance of these findings we measured the levels of IgG and IgM produced in culture. Decreased levels of IgG (Fig. 9d) and IgM (Fig. 9e) were evident in supernatants obtained, at 7 days, from co-cultures of PBMCs with HIV-1 exposed FDCs as compared to PBMCs cultured with control FDCs.

## Discussion

In spite of successful anti-retroviral therapy (ART), and in presence of undetectable viremia in blood, several studies have shown that HIV-1 structural proteins and glycoproteins persist within the GCs of HIV-1 infected patients; it has been suggested that these viral proteins may be present in ICs captured by FDCs and be involved in the maintenance of long-term humoral immunity to HIV-1 in absence of viral replication [28]. When examining post-mortem material from HIV-1 infected subjects Keele and collaborators [29] showed that HIV-1 virus trapped on FDCs was infectious and genetically diverse. The authors also emphasized the relevance of FDCs as reservoirs for HIV-1 infection as, in comparison to other cell types which are currently considered as reservoirs for HIV-1 infection, FDCs can trap a large amount of intact virus particles which can be directly transmitted to HIV-1 target cells. These virus particles are maintained at the surface of FDCs by HIV-1 antibody captured through the presence of high levels of thiol groups and sequestration of virions in dendritic processes of FDCs. The virus complexed at the surface of FDCs can be transmitted to several cell types, including T cells and macrophages, the latter event taking place through an intercellular communication network controlled by the P-selectin glycoprotein ligand 1 [30].

FDCs are stromal cells located in B cell follicles. They have the ability to trap immune complexes and rescue B cells from apoptosis. FDC are CD45-negative, express CD21, CD23, CD35, CD40, CD44, CD73, ICAM-1, VCAM-1, BAFF and MFGE8, and secrete CXCL13 [4, 7]. All these characteristics were found in our FDC lines as shown in the present paper or in our previous articles [4, 27, 31]. We also compared FDC lines with fresh isolated FDC, and found equivalent antigen phenotypes and functions in both types of cells [31]. The only exception was the expression of CD35, which has been detected in fully mature FDC, but was not found in our FDC lines (this work). This difference suggests that FDC lines consist of precursors of FDC (pre-FDC) similar to those reported by Krautler et al. in the mouse [3]. Like our FDC lines, these murine pre-FDCs are CD140b+ (PDGFβ) and express alpha smooth muscle actin-ASMA [this paper (4, 27, 31)]. Although our FDC lines are pre-FDC, they display typical FDC functions such as immune complex trapping and inhibition of B cell apoptosis [31].

Studies assessing in vitro the infection of FDCs by HIV-1 have not been previously performed; this is due to the fact that FDCs are difficult to isolate from lymphoid tissue as they represent only 1 % of the cells within this compartment [4]. Upon these conditions FDC lines were characterized and shown to share morphological and functional characteristics with bone marrow stromal cell progenitors and myofibroblasts. Human FDCs have also been shown to share similarities with decidual stromal cells (DSCs) as both cell types display supportive activity for B cells. A recent study also demonstrated that FDCs and DSCs showed distinctive features as supporting cells for B lymphocytes: both cell types produce BAFF and CXCL13 and have the capacity to rescue B cells from apoptotic processes [27]. In addition it was shown that both FDCs and DSCs are related to mesenchymal stem cells.

Following the many evidences suggesting that HIV-1 particles do not replicate in FDCs, but carry the virus in form of ICs, it has been suggested that FDCs may contribute to late phases of persistent viremia during ART in infected patients [32], an event mediated in part by the low penetration of ART into lymphatic tissues and allowing for persistent virus replication [33].

In our experiments, the exposure of FDC primary lines to HIV-1 isolates did not change the expression of molecules which have previously been used to characterize the lineage specificity of these cells [4]. The cells expressed low levels of CD4 and of potential HIV-1 receptors including CD21, Siglec 1, CXCR4 and members of the TAM receptor family including TAM Axl, TAM Mer and Dtk Mer, which have been suggested as alternative receptors for the adhesion of HIV-1 to target cells. In order to dissect if the multitude of potential HIV-1 receptors expressed on the surface of FDCs play a role for HIV-1 attachment further studies are needed. It is also possible that selected populations of FDCs within the FDCs lines used in our experiments may display a different degree of sensitivity to HIV-1 attachment and infection.

In our experiments, minimal levels of the p24 protein could be detected in supernatants from HIV-1 exposed FDC cultures; it was however not possible to dissect whether the presence of this HIV-1 protein in virus supernatants could be due to virus production or to trapping of virus particles from the initial virus inoculum at the surface of HIV-1 exposed FDCs. It is however unlikely that, in absence of specific HIV-1 antibodies, the virus may persist for several days trapped at the surface of FDCs.

As by interacting with B and T cells FDCs play a pivotal role for the establishment of GCs, we performed experiments to clarify whether exposure of FDCs to HIV-1 perturbates functions which may be pivotal for the interaction between FDCs and B cells including chemotaxis and survival. Chemokines are important molecules to mediate chemotaxis of cells to functional niches; by using microarray and qPCR we could show that the exposure of FDCs to distinct HIV-1 isolates increased the gene expression of a number of distinct chemokines which are important for migration of macrophages and neutrophils (CXCL3, CXCL5, CXCL6). However the gene expression of chemokines important for attraction of T and B cells to FDCs was not altered by the exposure of FDCs to HIV-1 isolates. This was also confirmed by the equal amount of T and B cells, migrating towards cell culture supernatants of HIV-1 exposed FDCs following cell activation through different stimuli. Accordingly, the exposure of FDC lines to HIV-1 did not lead to changes in chemo-attraction of T and B cells.

The microarray experiments revealed an increased expression of the inflammatory cytokine IL-8 in HIV-1 exposed FDCs. The increased gene expression of the inflammation mediator IL-8 prompted us to measure the levels of additional cytokines in the supernatants of HIV-1 exposed and non-exposed FDC lines.

Interestingly the levels of several inflammatory cytokines including IL-1β, GM-CSF, IL-6 and IL-8 were produced to a much larger extent from FDCs exposed to virus strains HIV-1 IIIB and SF162 than control cells. The production of these inflammatory cytokines may lead to various biological mechanisms in cells interacting with FDCs in vitro. Increased levels of GM-CSF may accelerate the differentiation of DCs and macrophages locally and perturbate the correct differentiation program in these cells. Experiments conducted to assess whether the production of inflammatory cytokines could be mediated by virus components, in addition to native virus strains, revealed that heat-inactivated virus was as efficient as intact virus in inducing production of the inflammatory cytokines IL-6 and IL-8. The molecular mechanism involved in this pathological effect is not clarified but it will be important to dissect whether binding of HIV-1 surface protein gp120 to potential HIV-1 receptors has a role for inducing production of inflammatory cytokines from FDC lines. According to the results presented by us it is likely that virus captured at the surface of FDCs in the GCs may act to induce production of inflammatory cytokines thus affecting B cells in the close proximity.

Incubation of HIV-1 exposed FDCs with B cells resulted in a consistent depletion of B cells in the co-cultures. It is unlikely that HIV-1 upon these conditions may infect B cells as these cells do not express HIV-1 receptors. It was recently proposed that pyroptosis, a form of cell death, could lead to depletion of CD4+ T cells during HIV-1 infection [34]. Pyroptosis takes place during intense inflammatory conditions mediated by pre-inflammatory cytokines, including IL-1β, a cytokine which we could detect in supernatants of HIV-1 exposed FDCs. It is possible that the high concentration of several inflammatory conditions detected in the supernatants of HIV-1 exposed FDC cultures may affect the survival of B cells present in co-culture. It is puzzling that B cells may be affected upon this condition without any detected effect on T cells present in culture. It is however important to point-out that a reduction of IgG and IgM production took place in co-cultures of B cells with HIV-1 exposed FDCs; accordingly the declined number of B cells detected upon these conditions translates into declined production of Igs and may reflect mechanisms operating in vivo and responsible for reduced levels of serological memory.

## Conclusions

In conclusion we describe that exposure of FDCs to HIV-1, although not leading to clearly detectable virus production, results in the increased expression and production of inflammatory cytokines. The inflammatory environment which develops upon these conditions results in impaired survival of B cells. This in vitro model suggests that a similar mechanism may take place in vivo in the germinal center and result in damage of B cells thus contributing to impaired B cell responses during HIV-1 infection.

## Methods

### Isolation and culture of FDC lines

FDC lines were established as previously described [4]. Tonsil samples were obtained from patients with tonsillitis at the Hospital Universitario Virgen de las Nieves (Granada, Spain). The patients (3–10 years old) were in complete remission before tonsillectomy. Informed consent was obtained from the parents or guardians of each patient. This study was approved by the Comité Ético y de Investigación (Ethics and Research Committee) of the Hospital Universitario Virgen de las Nieves.

Human tonsils were thoroughly washed in PBS solution and cut into small pieces that were finely minced between two scalpels in a small volume of RPMI 1640 medium supplemented with 100 U/ml penicillin and 50 μg/ml gentamicin. In order to increase the release of FDCs from tissue, the cell suspension was exposed to 0.25 % trypsin and 0.5 mM EDTA (Sigma-Aldrich) for 15 min at 37 °C, and the enzymatic reaction stopped by adding cold RPMI 1640 with 20 % fetal calf serum (FCS). The suspension was filtered through gauze and centrifuged at 425×*g* for 10 min; the supernatant was discarded and the cell pellet was resuspended in RPMI 1640. After centrifugation of the cell suspension on Ficoll-Paque (Pharmacia Biotech) for 20 min at 600×*g*, cells were collected from the interface, resuspended in PBS and washed. Cells were cultured for 1 h at 37 °C in complete RPMI 1640 supplemented with 10 % FCS to allow macrophages and granulocytes to adhere to the flask. Thereafter, cells in suspension were washed and incubated in Opti-MEM (minimum essential medium; Invitrogen, Grand Island, NY) supplemented with 3 % fetal calf serum (FCS), 100 UI/ml penicillin, 100 µg/ml streptomycin and 0.25 mg/ml amphotericin (Sigma-Aldrich). After overnight incubation to allow adherent cells to attach to the flask, lymphocytes in the supernatant were discarded. The medium was then replaced and changed twice a week. After 2–4 weeks, adherent cells were morphologically uniform and covered the whole surface of the 25 cm^2^ culture flask. Cells viability was determined by trypan blue exclusion before any experiments were done. Only preparations with >95 % viable cells were used. Six cell lines: 1401, 1402, 1403, 8–13, 9–13 and 10–13 were used in different sections of this study. FDC lines were kept up to 15 in vitro passages. As the lines showed similar phenotypical characteristics prior and after infection, different cell lines were used for the different experiments.

### Characterization of FDCs by flow cytometry

FDCs were detached from the culture flask by treatment with Trypsin/EDTA at room temperature for 5 min. Thereafter the cells were washed once with 5 % FCS/PBS, resuspended at a density of 10^6^ cells/ml in 5 % FCS/PBS and incubated with the appropriate mAb for 30 min at 4 °C in the dark. After washings the cells were resuspended in 0.2 ml 2 % Paraformaldehyde (PFA)/PBS and immediately analyzed in a flow cytometer (Becton Dickinson, Mountain View, CA, USA) using Cellquest software (Becton Dickinson).

The percentage of cells that were positive for the different antigens was calculated by comparison with the appropriate isotype control. For indirect labeling, FITC-labeled goat anti-mouse Ig was added after the first mAb. Intracytoplasmic labeling was conducted by fixing the FDCs with PermFix (BD Bioscience) for 20 min at 4 °C; thereafter the cells were washed with 0.2 ml PermWash (BD Bioscience) before adding the mAbs. All analyses were evaluated with a FACScan flow cytometer.

The monoclonal antibodies (mAbs) used to characterize the phenotypical characteristics of FDCs were PE conjugated anti-CD10, PE anti-CD14, PE anti-CD21, FITC anti-CD31, FITC anti-CD35, APC anti-CD44, PE anti-CD45, FITC anti-CD54, Alexa Fluor 647 anti-Podoplanin, PE anti-HLA-DR all from BioLegend (San Diego, CA USA); FITC conjugated anti-CD90, PE anti-CD106, PE anti-CD140b, APC anti-CD146 and APC anti-Siglec-1 (CD169) were all from eBioscience (San Diego, CA USA). PE conjugated anti-MFGE8, APC anti-CXCL12 and APC anti-CXCL13, Alexa Fluor 488 anti-TAM AXI, APC anti-TAM Mer and PE anti-Dtk Mer were purchased from R&D Bioscience (Minnesota, USA) while APC anti-CCR5, PE anti-CXCR4, FITC anti-CD4, APC anti-DC-SIGN (CD209), APC anti-Integrin β7, PE anti-α4 (CD49d) and APC anti-CCR5 were from BD Bioscience (USA). The FITC conjugated anti-ASMA antibody was from SIGMA while the unlabeled anti-CD105 (clone P4A4) was a gift from Dr. F.J. Blanco (CIBER de Enfermedades Raras, Madrid, Spain). Matched isotype control antibodies were included in the stainings. The epitopes detected by these mAbs were not affected by the trypsin treatment used to detach the cells.

The Alexa Fluor 488 conjugated ovalbumin for antigen capture capacity test was from Life Technologies (address USA).

### HIV-1 viral stocks and infection

The HIV-1 strains IIIB (CXCR4 user) and SF162 (CCR5 user) were grown in PHA/IL-2 stimulated lymphocytes and clarified supernatant stocks were frozen at −80 °C. HIV-1 RNA load was measured by Cobas Amplicor (Roche Molecular Systems Inc., Branchburg, New Jersey, USA) and was 150 × 10^6^ copies/ml for both viruses. FDC cultures at about 70 % confluence were washed once with PBS and exposed to 1 ml of IIIB or SF162 overnight; cells were thereafter washed two times with PBS to remove unabsorbed viruses. Fresh OPTI medium from Gibco supplemented with 3 % FCS, 50 U/ml penicillin and streptomycin was then added to the cells and cultures protracted according to the experiments to be conducted. For co-culture with PBMCs, FDCs were cultured in 6-well plates and exposed to HIV-1 as described above for 2 days. The IIIB virus used for infection and its mock preparations were assessed for the contents of cytokines GMCSF, IL-1β, IL-6 and IL-10 with commercial ELISA kits (R&D Minneapolis, USA); the concentration of these cytokines did not exceed 0.5 ng/ml.

Blood from healthy donors was the source of PBMCs which were prepared by gradient centrifugation and cultured in RPMI 1640 containing 10 % FCS, 50 U/ml penicillin and 50 U/ml streptomycin (HyClonem Thermo scientific, Logan, USA). PBMCs were added to FDC cultures at the density of 2 × 10^6^ cells/well. PBMCs were collected after one week co-cultivation with HIV-1 exposed or non-exposed FDCs and the viability and frequency of T and B cells were measured.

LIVE/DEAD^®^ Fixable Near-IR Dead Cell Stain Kit from Life Technologies was used to determine viability of PBMCs. APC conjugated anti-CD3 and PE anti-CD19 monoclonal antibodies (BD Bioscience) were used for monitoring the frequency of living T and B cells. Cells were acquired using LSR II Flow Cytometer (Becton Dickinson, San Jose, CA, USA) and analyzed through Flow-Jo software version 887 (Three Star Inc., OR, USA).

For detection of intracellular p24 HIV-1 protein in FDC cell lines exposed to either SF162 or IIIB strains, the anti-p24 antibody KC57-RD1 (Beckman Coulter, Brea, CA, USA) was used. Briefly, at 1 week post virus exposure to the virus the FDC cells were fixed with 4 % PFA and subsequently stained in Perm/Wash buffer (BD Biosciences, San Jose, CA, USA) for 2 h at +4 °C.

The Vironostika HIV-1 Ag/Ab Microelisa system (BioMerieux, France), based on a one-step “sandwich” principle, was used to detect production of the HIV-p24 antigen in supernatant of FDCs cells exposed to HIV-1.

### Determination of viral RNA and proviral DNA in FDC cells

FDCs were exposed overnight to HIV-1 IIIB and SF162 strains; thereafter the cells were extensively washed in PBS and cultured for additional 7 days until separate RNA and DNA preparations were initiated from virus-exposed and control cells. RNeasy lysis buffer was added to the cells for total RNA extraction using RNeasy Mini Kit (SA Biosciences, QIAGEN, CA, USA). RNA extraction was performed according to the manufacturer’s recommendation. For DNA preparation, FDCs were washed twice with PBS prior to lysis. The total DNA was obtained using QIAamp DNA Mini kit (SA Biosciences, QIAGEN, CA, USA) according to manufacturer’s instructions. RNA was also prepared from cell culture supernatants obtained at day 0 and day 7. Extracted RNA was used for cDNA synthesis using primer JA82 [35, 36] and Thermoscript (Life Technologies, Stockholm, Sweden) essentially according to the instructions of the manufacturer.

HIV-1 proviral DNA and cDNA was diluted in four tenfold-steps and PCR amplified in duplicate using HIV-1 pol primers JA79-JA82 and JA80-JA81, known to amplify diverse genetic subtypes within the HIV-1 group M [35, 36]. Lysates from HIV-1 LAI infected cells and dH_2_O were included as positive, respectively negative, control for each PCR running. Two independent PCRs were performed on two independent cell preparations. The amplicons were visualized by GelRed staining after electrophoresis in a 1.5 % agarose gel.

### PCR microarray for chemokine and chemokine receptors

Human chemokines and chemokine receptors PCR array (SA Biosciences, QIAGEN, CA, USA) used in this study is designed to analyze 84 chemokines and chemokines receptor genes; 4 housekeeping genes were used for normalization of the data.

Non-exposed FDC cells were compared to HIV-1 exposed cell cultures to study genes differentially expressed in HIV-1 IIIB and SF162 exposed FDCs. The PCR Array was performed according to manufacturer’s instructions. The results were analyzed using RT2 profiler PCR array data analysis version 3.5.

### Real-time PCR

Total RNA from the line FDC 10.13 and FDC 9.13 (control and HIV-1 exposed) was extracted as described, and cDNAs were synthesized. Primers were designed corresponding to the genes found dysregulated in the microarray experiments. Each primer was submitted into an NCBI BLAST search to ensure specificity for the target mRNA (Table 1). The primers were synthesized by Integrated DNA Technologies (San Diego, California, USA).

**Table 1:** Sequences of primers used for real-time PCR

Gene	Primers	
	Forward	Reverse
CCL2	AGCAGCAAGTGTCCCAAA	GAGTTTGGGTTTGCTTGTC
CCL25	TGGATGCTCGAAATAAGGTT	CATTCCTCTTACTGCTGCT
CXCL3	AAAGTGTGAATGTAAGGT	TAAGCTTCTTACTTCTCTCC
CXCL5	GGAAATTTGTCTTGATCCAG	AGACCTCCAGAAAACTTCTC
CXCL6	GCGTTGCACTTGTTTACGCG	CCAGAAAACTGCTCCGCTGA
IL-8	CCCAAATTTATCAAAGAACTGA	GCATCTTCACTGATTCTTGG
IL-4	AAGTGCGATATCACCTTACA	TCATGGTGGCTGTAGAACT

Real-time RT-PCR was performed under conditions of 2 min at 50 °C, followed by 10 min at 95 °C, 95 °C 1 min and 1 min at 58 °C (CXCL6), 52 °C (CCL2 and CCL25), 48 °C (CXCL3), 46 °C (IL-4 and IL-8) and 45 °C (CXCL5) respectively for 40 cycles. Data were analyzed using AB Applied Biosystems 7500 and 7500 Fats Real Time PCR Systems version 2.0.1 and were converted into threshold cycle (Ct) values. The samples were normalized by quantitation-relative curve with human beta-actin.

### Migration assay

Cell migration assay was conducted using 24-well insert (Costar) with 5-µm pores. PBMCs from normal, healthy donors were activated with either CPG-ODN, a synthetic TLR9 agonist (Invitrogen), or Phytohemagglutinin (PHA) (Sigma) or anti-human IgM (Jackson Immuno Research) and anti CD40 monoclonal antibody, clone B-B20 (BioSite).

The activation status of the cells was then investigated by measuring CD69 expression on CD19+ and CD3+ PBMCs by Flow cytometry prior to migration assay. After 2 days of activation, 0.5 × 10^6^ PBMCs were added in the upper insert of the migration plates whereas 0.6 ml of supernatant from HIV-1 exposed or non-exposed FDCs were added in the lower insert. After 4 h at 37 °C, the inserts were removed, the cells collected from the lower wells and counted in a hemacytometer. Thereafter, the migrated cells were fixed with 2 % PFA and the frequency of CD19 and CD3 positive cells among migrated cells was evaluated.

The monoclonal antibodies (mAbs) used to identify and characterize the PBMCs in the migration assay are PE anti-CD69, FITC and PE anti-CD19, APC and FITC anti-CD3, PE and FITC isotype antibodies all purchased from BD Bioscience.

### Antigen capture assay

HIV-1 exposed FDCs (10-13, 1402 and 1403) were cultured for 24 h at 37 °C and 5 % CO_2_. After removing the virus, cells were washed extensively with PBS and incubated in growth medium supplemented with 1 mg/ml Alexa Fluor 488 conjugated ovalbumin for 2 h at 37 °C and 5 % CO_2_. Finally cells were washed three times, detached from the culture plate by Trypsin–EDTA for 5 min and immediately analyzed in a FACSCalibur flow cytometer.

### Analysis of culture supernatants

The IgG and IgM production in 7 days’ co-cultures of HIV-1 exposed FDCs with PBMCs was measured in culture supernatants by ELISA. IgG production was analyzed in culture supernatants by an in-house ELISA, using a goat anti-human IgG (H + L) (Thermo Fisher, Waltham, MA) as capture antibody, a goat anti-human IgG conjugated with Horse-Radish-Peroxidase (HRP) (Thermo fisher, Waltham, MA) as a secondary antibody and the TMB substrate solution (BD Pharmingen). IgM levels were analyzed using ELISA a commercial kit from Mabtech (Nacka Strand, Sweden).

Supernatants of FDC lines exposed to HIV-1 and cultured for 10 days were screened for 12 human cytokines (IL-1α, IL-1β, IL-2, IL-4, IL-6, IL-8, IL-10, IL-12, IL-17A, IFN-γ, TNF-α, GM-CSF) using the Multi-Analyte ELISArray kit (Qiagen) according to the company’s recommendation. Virus strains IIIB and SF162 inactivated at 56 °C for 60 min were used to assess the relevance of virus replication in FDC lines for production of cytokines IL-6 and IL-8.

## Additional files

10.1186/s12977-016-0295-4 Gaiting strategy for detection of FDC cells positive for surface markers in cultures exposed or not to HIV-1 strains.
10.1186/s12977-016-0295-4 Gating strategy for detection of HIV-1 receptors CD4 and CCR5 in FDC lines.
10.1186/s12977-016-0295-4 Activation of B and T cells upon different stimuli. The frequency of activated CD69 + cells among PBMCs (A), B cells (B) and T cells (C) are shown when PBMCs were exposed to different activation stimuli.
10.1186/s12977-016-0295-4 Antigen capturing capacity of HIV-1 exposed FDCs. FDC lines (10-13, 1402 and 1403) exposed to HIV-1 IIIB or SF162 for 24 h were treated with 1 mg/ml Alexa Fluor 488 ovalbumin conjugated for 2 h. The binding of ovalbumin to FDCs was evaluated by FACSCalibur flow cytometer. The bars represent the mean and SD of relative fluorescence intensity (MFI) of ovalbumin binding in 3 HIV-1 exposed FDCs or control cells in relation to cells cultured in medium.
