# Supplementary figures and images for: Impaired B cells survival upon production of inflammatory cytokines by HIV-1 exposed follicular dendritic cells
Source: Retrovirology. 2016 Sep 5;13(1):61. doi: 10.1186/s12977-016-0295-4 (PMC5011926; doi:10.1186/s12977-016-0295-4)

## Slide 1
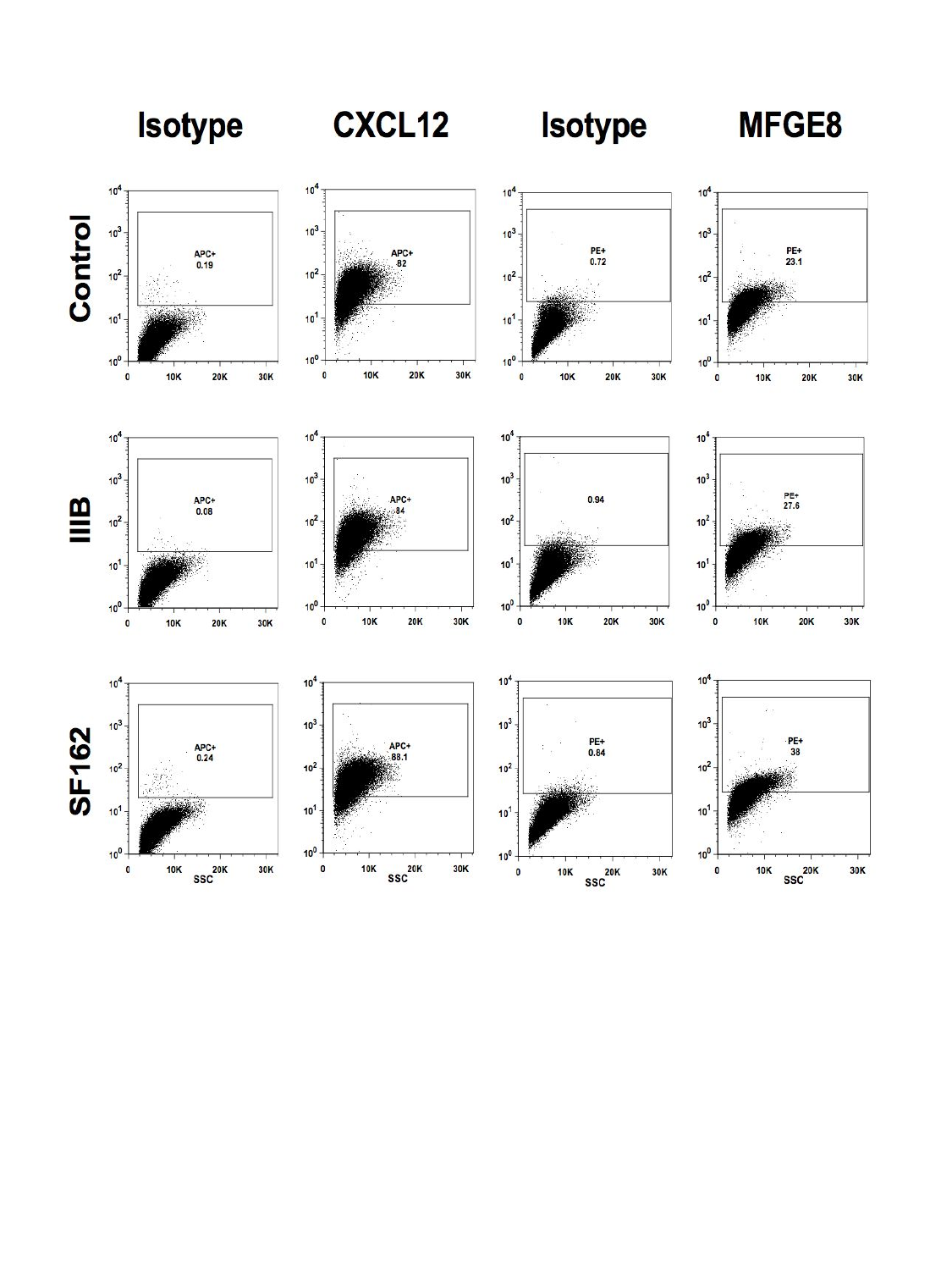

Supplement: Supplementary file 1 — 10.1186/s12977-016-0295-4 Gaiting strategy for detection of FDC cells positive for surface markers in cultures exposed or not to HIV-1 strains. [file 12977_2016_295_MOESM1_ESM.ppt]

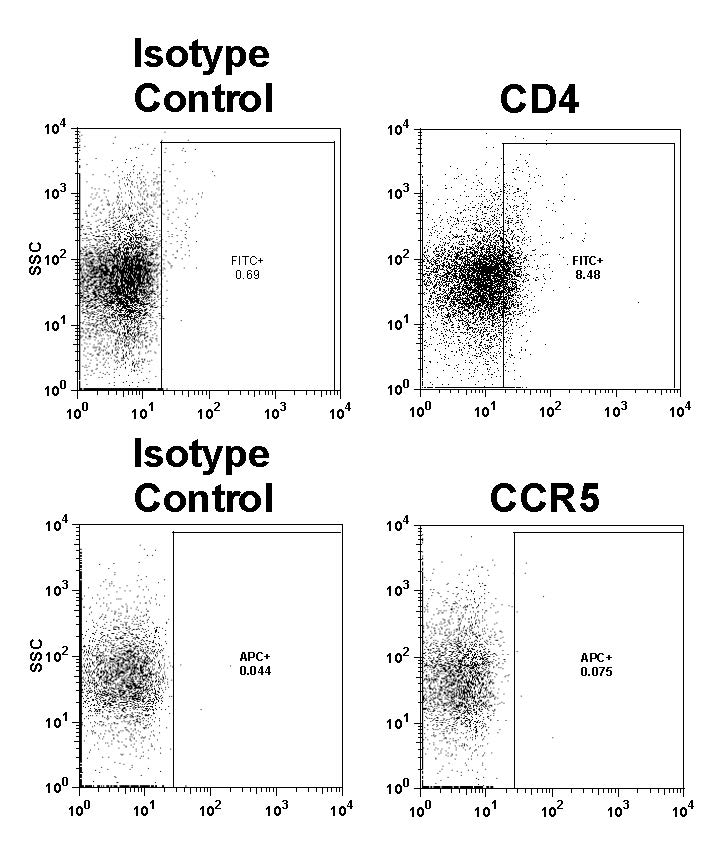

Supplement: Supplementary file 2 — 10.1186/s12977-016-0295-4 Gating strategy for detection of HIV-1 receptors CD4 and CCR5 in FDC lines. [file 12977_2016_295_MOESM2_ESM.jpg]

## Slide 1
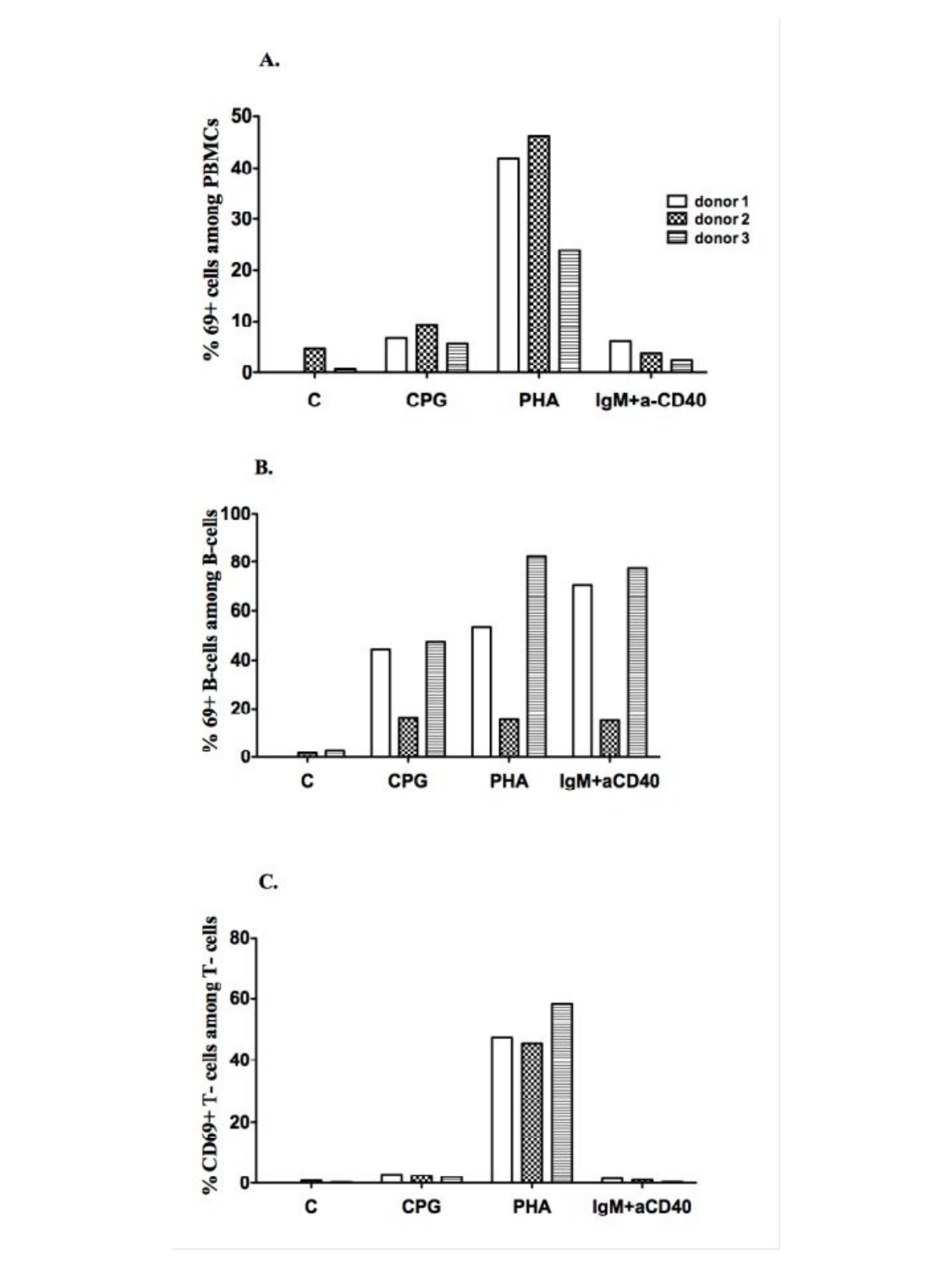

Supplement: Supplementary file 3 — 10.1186/s12977-016-0295-4 Activation of B and T cells upon different stimuli. The frequency of activated CD69 + cells among PBMCs (A), B cells (B) and T cells (C) are shown when PBMCs were exposed to different activation stimuli. [file 12977_2016_295_MOESM3_ESM.ppt]

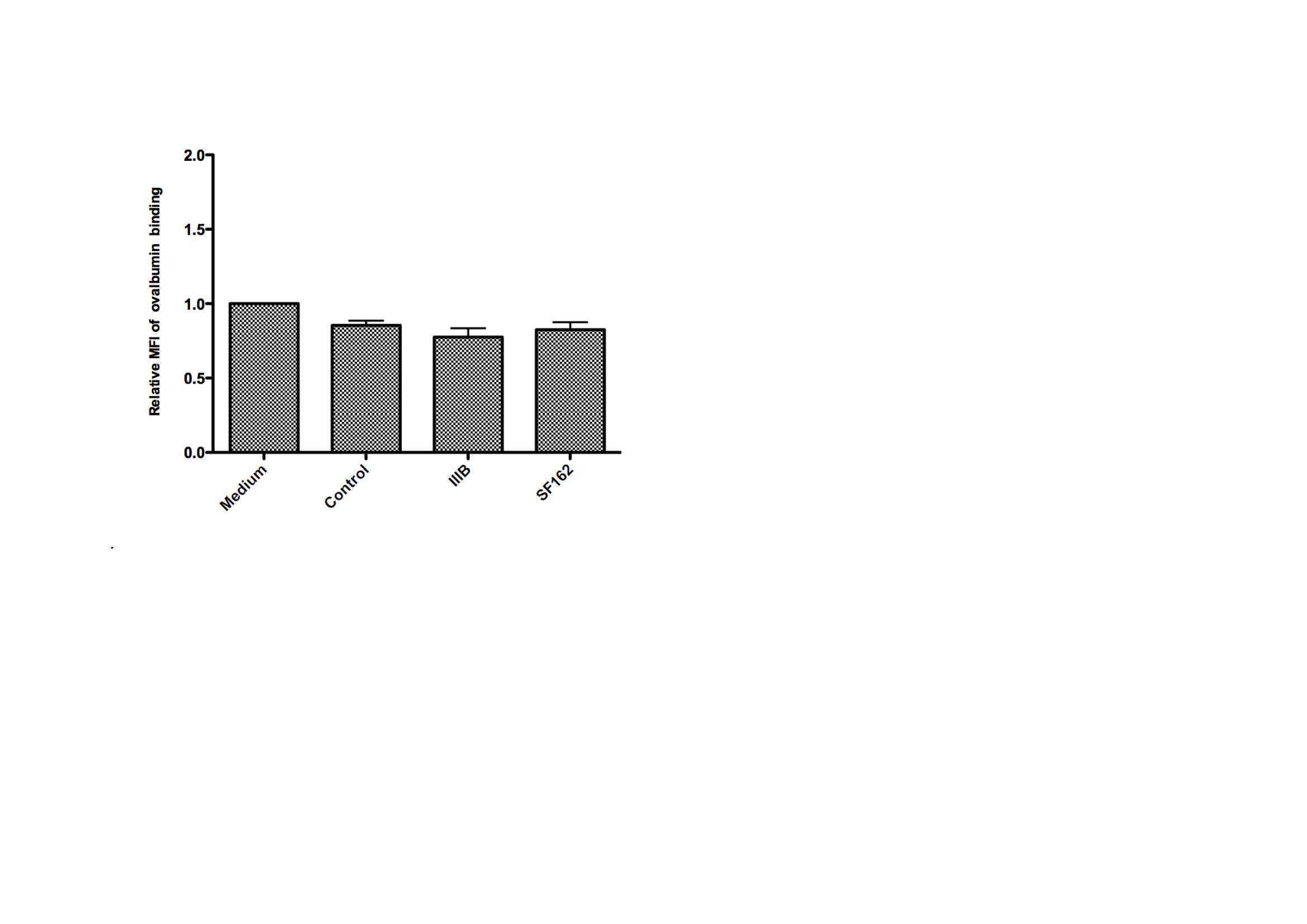

Supplement: Supplementary file 4 — 10.1186/s12977-016-0295-4 Antigen capturing capacity of HIV-1 exposed FDCs. FDC lines (10-13, 1402 and 1403) exposed to HIV-1 IIIB or SF162 for 24 h were treated with 1 mg/ml Alexa Fluor 488 ovalbumin conjugated for 2 h. The binding of ovalbumin to FDCs was evaluated by FACSCalibur flow cytometer. The bars represent the mean and SD of relative fluorescence intensity (MFI) of ovalbumin binding in 3 HIV-1 exposed FDCs or control cells in relation to cells cultured in medium. [file 12977_2016_295_MOESM4_ESM.jpg]
